# Postglacial recolonizations, watershed crossings and human translocations shape the distribution of chub lineages around the Swiss Alps

**DOI:** 10.1186/s12862-016-0750-9

**Published:** 2016-09-09

**Authors:** Alexandre Gouskov, Christoph Vorburger

**Affiliations:** 1 Eawag, Aquatic Ecology Department, Überlandstrasse 133, P.O.Box 611, 8600 Dübendorf, Switzerland; 2 Institute of Integrative Biology, ETH Zürich, Universitätstrasse 16, 8092 Zürich, Switzerland

**Keywords:** Chub, Drainage capture, Human translocations, Phylogeography, Pleistocene glaciations, *Squalius*

## Abstract

**Background:**

Distributions of European fish species were shaped by glaciations and the geological history of river networks until human activities partially abrogated the restrictions of biogeographical regions. The nearby origins of the Rhine, Rhone, Danube and Po rivers in the Swiss Alps allow the examination of historical and human-influenced patterns in fish genetic structure over a small geographic scale. We investigated these patterns in the widespread European chub (*Squalius cephalus*) from the Rhone, Rhine and Danube catchments and its proposed southern sister species Italian chub (*Squalius squalus*) from the Po catchment.

**Results:**

A phylogenetic tree constructed from mitochondrial Cytochrome b and COI sequences was consistent with earlier work in that it showed a separation of European chub and Italian chub, which was also reflected in microsatellite allele frequencies, morphological traits and shape differences quantified by geometric morphometrics. A new finding was that the predominant mitochondrial haplotype of European chub from the Rhine and Rhone catchments was also discovered in some individuals from Swiss populations of the Italian chub, presumably as a result of human translocation. Consistent with postglacial recolonizations from multiple refugia along the major rivers, the nuclear genetic structure of the European chub largely reflected drainage structure, but it was modified by watershed crossings between Rhine and Rhone near Lake Geneva as well as between Danube and Rhine near Lake Constance.

**Conclusion:**

Our study adds new insights into the cyprinid colonization history of central Europe by showing that multiple processes shaped the distribution of different chub lineages around the Swiss Alps. Interestingly, we find evidence that cross-catchment migration has been mediated by unusual geological events such as drainage captures or watershed crossings facilitated by retreating glaciers, as well as evidence that human transport has interfered with the historical distribution of these fish (European chub haplotypes present in the Italian chub). The desirable preservation of evolutionarily distinct lineages will thus require the prevention of further translocations.

**Electronic supplementary material:**

The online version of this article (doi:10.1186/s12862-016-0750-9) contains supplementary material, which is available to authorized users.

## Background

Strong climatic fluctuations during the Pleistocene induced extensive population contractions and expansions in the European flora and fauna [1]. The geographic situation of Europe, surrounded by sea and divided by east–west mountain barriers, repeatedly trapped species in glacial refuges until recolonization during the warm interglacial periods. The repeated separation into glacial refuges led to genetic subdivision in many species, with hybrid zones forming where they came into contact again following recolonization [2]. In freshwater fishes, populations are additionally constrained by the geological history of a region. They remain restricted to their hydrographic basins unless new interconnections or chance dispersal over land allow further expansion. Accordingly, the highest species diversity is found in the historically ice-free but isolated river catchments of Peri-Mediterranean and Ponto-Caspian Europe, and the lowest diversity in northern and central Europe ([3] and references therein).

Human activities have partially abrogated the borders between biogeographical regions. Today, shipping waterways such as the Rhine-Main-Danube canal or the Rhone-Rhine canal connect most important watersheds throughout Europe, and commercial and recreational fisheries spread species of interest either actively by stocking or accidentally by live bait release, increasing the potential for hybridization between related but geographically separated taxa. Indeed, non-native fish have become one of the major threats for native species of the Mediterranean region by enabling hybridization and introgression, but also by the transmission of novel parasites and diseases or competition and predation [4].

Here we investigate the potentially human-influenced current biogeography of European chub from the *Squalius cephalus* complex in Central Europe, with a particular focus on Switzerland, where four major river systems originate in close proximity (Rhine, Rhone, Danube and Po). The European chub originated during the Pliocene (3–2.5 Myr ago) in the Tigris-Euphrates basin, where secondary contacts and hybridization with other *Squalius* species of previous waves of colonization played an important role in the evolutionary history of the group [5]. The invasion was at the end of an invasion period of several fish genera with Asian origin, which lasted from the Oligocene to the Pliocene (44–2.5 Myr ago), composing the large majority of today’s European fish species [6]. A rapid radiation of the chub from its Mesopotamian origin led to four contemporary phylogroups that are represented by four major clades in a mitochondrial cytochrome b (Cyt b) phylogeny, namely a Western, Adriatic, Aegean and Eastern lineage [7, 8]. Collectively they cover almost the entire European continent [5]. Italian and Greek haplotypes found by Durand et al. [7] are frequently considered a separate species, the Italian chub *S. squalus* [9, 10], although this is not universally adopted [8]. Western Europe appears to have been colonized out of the Danubian basin before the last Würm glaciation, probably during the Riss - Würm interglacial period (about 100,000 years ago), and holocene recolonization of the Western lineage presumably started from multiple Würm refuges (Rhone, Rhine, Danube, and tributaries of the Black Sea) [7, 8].

**Fig. 1:**
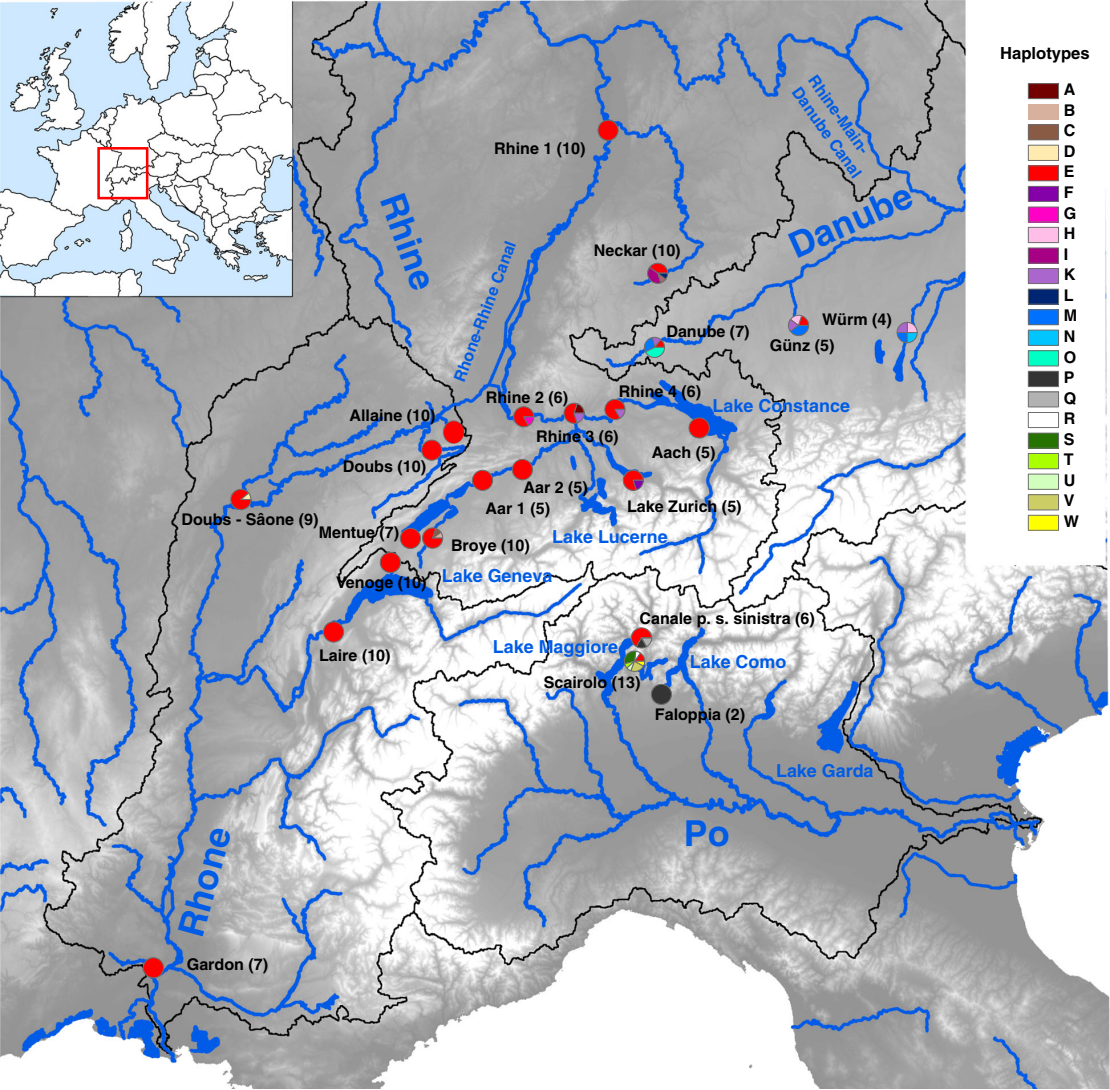
Overview of the study region. Sampling sites are represented by pie charts indicating the relative frequencies of mitochondrial haplotypes found at these sites. Sample sizes are in brackets. Catchments are outlined by black borders and shipping channels connecting catchments are annotated with their names in blue font. The small embedded map shows the location of the study site within Europe

Of particular relevance for the present study is the close proximity of the upper Danube catchment to the Rhine catchment in the area of Lake Constance, which was covered by the Rhine glacier during the Würm glaciation, and where meltwater streams and temporal proglacial lakes have facilitated exchange between the Rhine and Danube drainages during the glacier's retreat until current Lake Constance was formed and all its water drained west to the Rhine [11, 12]. A legacy of this exchange is still evident in the mitochondrial genetic diversity of perch (*Perca fluviatilis*) in Lake Constance [11]. Interestingly, there is an ongoing capture of the upper Danube drainage by the Rhine, such that water from the Danube deviates through the karst underground towards the Rhine catchment in the so-called Danube Sinkhole [13, 14]. A pecularity of the Rhone catchment is that below Lake Geneva it was disconnected from the upper catchment by a 60 m deep underground pass of the Rhone called “Pertes du Rhône”, but this natural spectacle was flooded in 1948 after construction of a dam for hydroelectric power production (Lake Genissiat). Finally, the different catchments were connected for shipping by the Rhone-Rhine Canal in 1833 and the Rhine-Main-Danube Canal in 1981 (Fig. 1). This situation provides an opportunity to study how natural and anthropogenic processes shaped the current distribution of chub lineages around the European Alps. We do this by applying genetic analyses of mitochondrial gene sequences and nuclear microsatellites as well as morphological analyses to chub from the four major river catchments originating in the Swiss Alps.

## Results

### Mitochondrial genetic variation

**Fig. 2:**
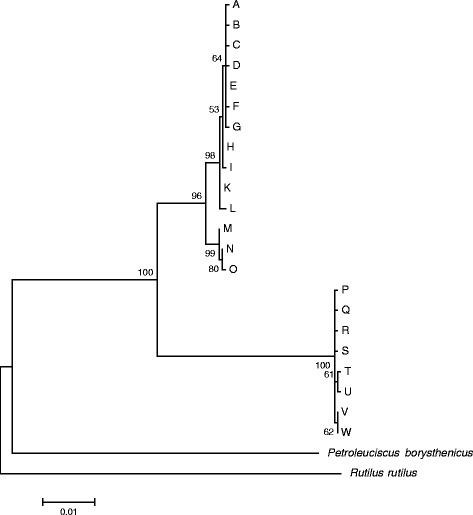
Maximum likelihood phylogenetic tree of chub haplotypes obtained from concatenated mitochondrial *COI* and *Cyt b* sequences, based on the Tamura-Nei model [54]. *Petroleuciscus borysthenicus* and *Rutilus rutilus* are used as outgroups. Letters A to O are European chub (*Squalius cephalus*) haplotypes and P to W are from Italian chub (*S. squalus*). The tree is scaled with branch lengths indicating amino acid substitutions per site (see scale bar). Numbers at nodes represent percent bootstrap support from 1000 replications. Lower than 50 % support values are not shown

Based on the concatenated COI and Cyt b sequences, 22 different haplotypes could be distinguished among the 168 chub analyzed. These haplotypes fell into two distinct monophyletic groups (Fig. 2). Haplotypes P-W all came from fish captured in the Swiss canton of Ticino, that is in putative Italian chub (*S. squalus*) from the Po catchment south of the Alps (Fig. 1). The other clade consisted of haplotypes A-O, which all represented European chub (*S. cephalus*) from the Rhine, Rhone and Danube catchments (Fig. 1), with one interesting exception (see below). A minor split within this clade separates haplotypes A-L from the three haplotypes M-O, which are restricted to the Danube catchment (Figs. 1 and 2).

The distinct status of Italian chub from the Po catchment was also supported by quantifying sequence divergence at the barcoding gene COI only. In total we could distinguish 8 COI haplotypes that fell into the same two major clades with a sequence divergence of 3.8 % between clades (Kimura’s two-parameter model [15]) and a mean sequence divergence of only 0.5 % within European chub sequences and 0.2 % within Italian chub sequences. Separate phylogenies for the two mitochondrial gene sequences are provided in Additional file 1: Figure S1.

Of the European chub haplotypes (A-K), haplotype E was by far the most common. It was the predominant haplotype in the Rhine catchment, virtually the only haplotype present in the Rhone catchment, where only one European chub out of 56 had a different haplotype (D), and it was also found in two chub from the Danube catchment (Fig. 1). Surprisingly, haplotype E was also discovered in some Italian chub from two sites south of the Alps in the Po catchment (Fig. 1). This is the only haplotype shared between European and Italian chub, which are otherwise clearly distinct mitochondrially (Fig. 2).

### Nuclear genetic variation

Two microsatellite loci, LC 128 and LceCb, could not be amplified in 14 out of the 20 individuals we genotyped from the Po drainage. Thus, all following analyses were restricted to eight loci that could be amplified in all 167 individuals and did not show any evidence of null alleles (see Additional file 2: Table S1 for sample sizes per site). These loci had an average of 15.6 alleles, ranging from 2–42 alleles per locus. Because only few fish per sampling site were genotyped, we pooled all individuals within catchments, thereby ignoring any substructure within catchments and possibly violating assumptions of genetic equilibria. While there was no evidence for linkage disequilibrium in any catchment nor globally (no significant *P*-values after sequential Bonferroni correction), there was indeed significant homozygote excess when averaged across loci in the Rhine catchment (*P* = 0.047) and in the Rhone catchment (*P* = 0.002), presumably reflecting a Wahlund effect due to within-catchment substructure (see below). Homozygote excess was not significant in the Danube catchment (*P* = 0.617) and in the Po catchment (*P* = 0.092). Mean expected and observed heterozygosities across loci as well as allelic richness (*AR*) for the four drainages are listed in Table 1. Most notable is the comparatively low *AR* observed in the Rhine catchment, but as mentioned above, these estimates lump several distinct sites with small sample size for each catchment and should therefore be interpreted cautiously.

**Table 1:** Genetic diversity measures for European chub (*Squalius cephalus*) from the Danube, Rhine and Rhone catchments, and for the Italian chub (*S. squalus*) from the Po catchment

Catchment	*H*_*O*_	*H*_*E*_	*AR*
Danube (n = 16)	0.77	0.76	7.43
Rhine (n = 74)	0.71	0.74	5.23
Rhone (n = 57)	0.62	0.68	7.34
Po (*n* = 20)	0.64	0.68	7.83

*H*_o_ observed heterozygosity, *H*_e_ expected heterozygosity, *AR* allelic richness standardized for the smallest sample size (16)

Pairwise genetic differentiation expressed as *F*_ST_ was highly significant between all catchments and ranged from 0.05 (Danube-Rhine) to 0.19 (Rhone-Po) (Table 2). Highly significant differentiation among catchments was also observed when Jost’s estimator of differentiation *D* [16] was used, especially for chub from the Po catchment, which was differentiated with *D* = 0.44–0.52 from chub in the Rhine, Rhone and Danube drainages (Table 2).

**Table 2:** Genetic differentiation estimated as *F*_*ST*_ (above diagonal) and *D*_*est*_ (below diagonal) among chub from the Rhine, Danube and Rhone catchments (*Squalius cephalu*s) and the Po catchment (*S. squalus*)

	Danube	Rhine	Rhone	Po
Danube		0.05	0.14	0.09
Rhine	0.22		0.07	0.11
Rhone	0.38	0.18		0.19
Po	0.44	0.44	0.52	

All *F*_*ST*_ and *D*_*est*_ values are statistically significant at a Bonferroni-corrected significance level of 0.008 (all *p* < 0.001)

**Fig. 3:**
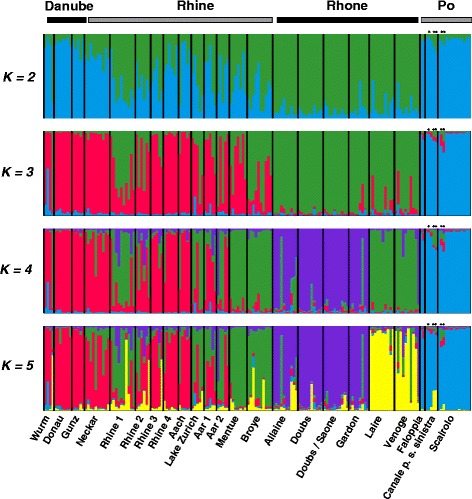
Results from the Bayesian clustering analysis in STRUCTURE using *K* = 2 to *K* = 5 clusters. Individuals are sorted by sampling site and the lengths of colored bars represent individual assignment probabilities to each of the inferred genetic clusters. Individuals from the Po drainage are Italian chub (*Squalius squalus*), all other individuals are European chub (*S. cephalus*). Italian chub that possessed mitochondrial haplotype E are marked with an asterisk

A more detailed picture of the genetic structure was provided by a Bayesian clustering analysis carried out with the software STRUCTURE [17]. Applying the method of Evanno et al. [18], the best-supported number of genetic clusters was somewhat ambiguous. The mean likelihoods *L*(*K*) showed a unimodal distribution with a maximum at *K* = 5, where Δ*K* also had a local maximum, but Δ*K* is also very high at *K* = 2 (actually slightly higher than at *K* = 5), even though mean *L*(*K*) for *K* = 2 is the lowest of all values tested (Additional file 3: Figure S2). Based on the combined evidence from *L*(*K*) and Δ*K* we conclude that *K* = 5 best reflects the number of genetic clusters, but we illustrate individual assignment probabilities for all values of *K* from 2 to 5 (Fig. 3). Under *K* = 5 many fish cannot be assigned to a single cluster with high probability, but some general patterns are nevertheless evident. Chub from the Po and the Danube catchments are mostly assigned to their separate clusters, chub from the Rhone catchment generally fall into two additional clusters, one of which comprises fish from just two rivers near Lake Geneva (Venoge and Laire), and the fifth cluster mainly comprises fish from the Rhine catchment, although many are assigned to this cluster with low probabilities only. Several chub from the Rhine catchment are even assigned to the cluster associated with the Danube catchment with high probability, indicating extensive admixture between these catchments (Fig. 3). To better understand the situation in the Rhone and Rhine catchments, it is instructive to also inspect individual assignment probabilities for values of *K* lower than the best-supported *K* = 5. Under *K* = 4, chub from the Rhone drainage remain assigned to two different clusters, but most fish from the Venoge and Laire near Lake Geneva are now assigned to the same cluster as most fish from the rivers Mentue and Broye, the two sites in the Rhine drainage that are geographically closest to Lake Geneva (Figs. 1 and 3). Chub from the Lake Geneva area thus appear more closely related to chub from the nearby parts of the upper Rhine catchment than to other chub from the Rhone catchment. For the Rhine catchment, the patterns under *K* = 4 show that particularly chub from sites close to Lake Constance (sites Rhine 3, Rhine 4 and Aach) and from the Neckar are assigned with high probabilities to the same cluster as the majority of chub from the Danube catchment. Under *K* = 3 chub from Rhine and Danube mostly fall into the same cluster, and under *K* = 2 chub from the Po catchment remain the most clearly defined cluster, to which most individuals from the Danube and many individuals from the Rhine are assigned with relatively high probabilities (Fig. 3).

Because the presence of the mitochondrial haplotype E in Italian chub from canton Ticino indicated the possible introduction of European chub in the Po catchment, we subjected all microsatellite genotypes to an analysis with the software NewHybrids 1.1 Beta3 [19], but none of the individuals were identified with any confidence as either F1or F2 hybrids or backcrosses.

### Morphological variation

A canonical variate analysis on size-corrected Procrustes coordinates revealed non-significant differences in shape among European chub from the Rhine, Rhone and Danube catchments, but clear and significant differences between Italian chub from the Po catchment and European chub from all other catchments (Table 3), although the difference between chub from Po and Rhine became non-significant after Bonferroni correction.

**Table 3:** Differences in body shape expressed as Procrustes distances (above diagonal) and associated *p*-values (below diagonal) among chub from the Danube, Rhine, Rhone (*Squalius cephalu*s) and Po catchments (*S. squalus*)

	Danube	Rhine	Rhone	Po
Danube		0.0136	0.0098	**0.0250**
Rhine	0.1168		0.0099	0.0181
Rhone	0.3531	0.0832		**0.0214**
Po	**0.0031**	0.0209	**0.0020**	

The differences between Po and Danube as well as Po and Rhone (in bold font) remain significant after Bonferroni correction (α = 0.008)

**Fig. 4:**
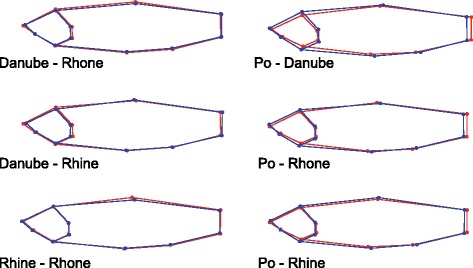
Pairwise shape differences between chub from different catchments, visualized by two-fold enhanced wireframe plots. Dots indicate landmark positions. For all comparisons, the mean shape of fish from the first catchment is colored in red and the mean shape of fish from the second catchment in blue

The pairwise shape differences between chub from the different catchments are illustrated in Fig. 4. Italian chub from the Po catchment have a more shallow and tapered head, more anterior insertions of the dorsal, pelvic and particularly the anal fins, and an elongated caudal peduncule compared to European chub (Fig. 4).

**Fig. 5:**
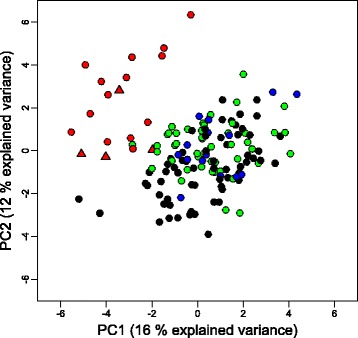
Positions of individual fish relative to the first and second principal component axes derived from the correlation matrix of traditional morphometric traits. Individuals are color-coded by drainage: green = Rhone, black = Rhine, blue = Danube (European chub, *Squalius cephalus*) and red = Po (Italian chub, *S. squalus*). Italian chub possessing the mitochondrial haplotype E are indicated by red triangles

Traditional morphometrics using size-standardized measurements of body parts, fin ray and scale counts as well as fin color produced a similar pattern in that European chub from the Rhine, Rhone and Danube catchments were very similar but distinguishable from the Po drainage’s Italian chub. The trait values for each catchment are summarized in Additional file 4: Table S2. Figure 5 illustrates the separation of Italian chub individuals from European chub in a plane described by the first two principal components of a PCA including all traits. The five traits contributing most strongly to the separation were the color of the fins (black in Italian chub, mostly red in European chub), the head length (shorter in Italian chub), the number of anal fin rays (more in Italian chub), the length of the anal fin (longer in Italian chub) and the snout length (shorter in Italian chub). There was no indication that individuals from the Po drainage possessing the European chub mitochondrial haplotype E were morphologically more similar to the European chub (Fig. 5). Because the first two PCs explained only 28 % of the total variance, we also compared chub morphology among catchments with MANOVAs on the PC scores from the first 14 PCs that cumulatively explain 80 % of the variance. These analyses confirmed that the largest morphological differences occur between European chub and Italian chub, but they also detected significant differences among European chub from different drainages (Table 4).

**Table 4:** Pairwise tests for differences in morphology between chub from the four drainages, based on MANOVAs on scores from the first 14 PCs (explaining 80 % of the variance) from a PCA on all traditional mophological traits (see Additional file 4: Table S2)

	Rhine	Rhone	Po
Danube	Pillai’s Trace = 0.33 *p* = 0.012	Pillai’s Trace = 0.46 *p* = 0.003	Pillai’s Trace = 0.91 *p* < 0.001
Rhine		Pillai’s Trace = 0.39 *p* = 0.003	Pillai’s Trace = 0.87 *p* < 0.001
Rhone			Pillai’s Trace = 0.90 *p* < 0.001

Pillai’s Trace and *p*-values are reported. All differences except the difference between chub from Danube and Rhine are significant at a Bonferroni-corrected significance level of 0.008

## Discussion

### Clear mitochondrial separation of European and Italian chub with evidence for human translocation

We observed a deep split between two major mitochondrial clades, one associated with chub from the Danube, Rhine and Rhone catchments (assigned to the Western lineage by Durand et al. [7]), and one associated with chub from the Po catchment (assigned to the Adriatic lineage by Durand et al. [7]). Sequence divergence between these clades at the DNA barcoding gene COI was 3.8 %, which corresponds to species-level divergence by commonly applied standards [20], although we acknowledge that gene trees can and should not be equated with species trees [21, 22]. The distinct status of chub from south of the Alps was also evident from the nuclear microsatellite data and from morphological variation. Two microsatellite loci did not amplify in most individuals from the Po catchment, suggesting high-frequency null alleles (i.e. mutations in the priming sites) at these loci, and the other loci showed strong differences in allele frequencies, resulting in highly significant genetic differentiation. The seemingly low *F*_ST_ values for such a deep split are a consequence of the dependency of *F*_ST_ on within-population diversity, which biases differentiation towards zero in highly polymorphic markers such as microsatellites [21]. Using Jost’s D [13], which does not suffer from this problem, estimates of genetic differentiation among catchments increased and were highest between the Italian chub from the Po catchment and European chub from the other catchments (Table 2), although the differentiation between European chub from the Rhone and Danube catchments was quite high as well. Italian chub also exhibited a distinct body shape (Fig. 4), and differed significantly from European chub in the ensemble of metric, meristic and qualitative morphological traits. Taking together the mitochondrial and nuclear genetic differentiation as well as the morphological differences we observed, our data do not contradict the treatment of *S. cephalus* and *S. squalus* as distinct species [9, 10], and for practical purposes, fin color remains the easiest trait to distinguish them. *Squalius squalus* reliably lacked the characteristic red hue present particularly on ventral and anal fins of *S. cephalus*. However, if *S. squalus* is given species status, the same might be warranted for chub belonging to the Aegean and Eastern lineages (*sensu* Durand et al. [7]), because at least mitochondrially, these are even more differentiated from the Western lineage [7, 8]. While distinct species names have been introduced for geographically restricted taxa that, according to [8], fall into the Aegaen lineage (e.g. *S. laietanus* for chub in northern Spain and southern France [23], or *S. orpheus* for chub in northern Greece and Bulgaria [24]), the mitochondrially most distinct chub from the Eastern lineage remain part of *S. cephalus* in the 'Handbook of European Freshwater Fishes' [25], which appears inconsistent. Thus we agree with Seifertova et al. [8] that a systematic revision of the *S. cephalus* complex is urgently needed.

Given the clear differences between European and Italian chub and the deep split between the two mitochondrial haplogroups, we were surprised to find mitochondrial haplotype E, the dominant haplotype in European chub from the Rhine and Rhone catchments, in some Italian chub from canton Ticino (in 6 individuals out of 21). It is theoretically possible that this reflects incomplete lineage sorting [26], i.e. that we recovered a mitochondrial polymorphism that pre-dates the evolutionary split between European and Italian chub and has persisted in the Italian chub. However, we do not consider this explanation likely because despite considerable haplotype diversity in both mitochondrial clades (Fig. 2), we did not discover some additional haplotype related to the European chub haplogroup in Italian chub from canton Ticino, we discovered a haplotype that is an exact match to the dominant haplotype in European chub of the geographically closest European chub populations from the Rhine and Rhone catchments (Fig. 1). Also, earlier studies did not observe any shared haplotypes between chub from the Po drainage and chub from north of the Alps [7, 8]. We consider it more likely, therefore, that this finding reflects a mitochondrial introgression after human-mediated transport across the Alps. Although the European chub is not comprised in the list of 38 introduced fish species in Italy [27], unrecorded releases cannot be excluded, and because our study region in the Swiss canton of Ticino is well-frequented by tourists, translocation as live fishing bait is a likely route of introduction. It is maybe suggestive that four Italian chub possessing the E haplotype showed somewhat more signs of nuclear admixture than other Italian chub in our Bayesian clustering analysis (Fig. 3), yet the analysis with the software NewHybrids detected neither first or second generation hybrids nor backcrosses. That does not exclude the possibility of nuclear admixture, however, because our power to detect hybrid individuals was very limited with only 8 loci. For efficient detection of F1 hybrids, 12–24 loci are recommended [28], even more are required for reliable detection of backcrosses, and the additional substructure among European chub from different catchments could have additionaly complicated hybrid detection. Furthermore, the suspected translocations may well be less recent. Introgression from the European chub has already been reported in the Catalan chub (*S. laietanus*) in southern France [29]. Depending on how frequently it occurs, such introgression may also be of concern for the lineage integrity of the Italian chub in Switzerland, where it has recently been taken up in the fisheries legislation as a distinct species, in accordance with the modification of the IUCN species list [30].

### Evidence of watershed crossings in European chub

For the chub's colonization of Europe north of the Alps, a two-step scenario was proposed by Durand et al. [7] and Seifertova et al. [8]: An initial Pleistocene colonization via the Danube, and a rapid post-Pleistocene expansion into Rhine and Rhone from western refugia following the last glaciation. Our results are consistent with this scenario. The Rhine and Rhone populations share the same dominant mitochondrial haplotype and possess very low mitochondrial diversity compared to European chub from the Danube, the nuclear genetic structure largely reflects drainage structure, and especially the chub from the Rhine catchment also exhibit comparatively low allelic richness (*AR*) at nuclear microsatellite loci (Table 1). Because *AR* is more sensitive than heterozygosity to founder effects [31], this is suggestive of a recent population expansion in this drainage.

However, the Bayesian clustering analysis of microsatellite genotypes also provided interesting evidence of genetic exchange between catchments. Firstly, chub from the Lake Geneva area of the Rhone catchment were assigned to a separate cluster and seemed more closely related to chub from the nearby parts of the upper Rhine catchment than to other chub from the Rhone catchment. This pattern is remarkable because it is also observed in the brown trout, *Salmo trutta* [32] in the European grayling, *Thymallus thymallus* [32] and in the bullhead, *Cottus gobio* [32, 33]. It has been explained by a postglacial watershed crossing, facilitated by the retreating Rhone glacier after the Würm glaciation [33]. It appears that the same process has also led to the colonization of Lake Geneva by chub from the Rhine catchment, which is the first example of this for a cyprinid fish for this region. These chub remained to some extent distinct because upstream colonization from the lower Rhone was impeded by the “Pertes du Rhône” that was only submerged since the construction of Lake Genissiat in 1948 (see Background).

Secondly, many chub from the Rhine catchment are assigned to the Danube cluster with high probability, indicating extensive admixture between these two catchments. This admixture may reflect the historical opportunity for genetic exchange between the two catchments during the last retreat of the Rhine glacier (see Background), it may be mediated by the extant drainage capture via the Danube sinkhole, or nowadays it may also be facilitated by the Rhine-Main-Danube canal. Considering that admixture is most evident in the sites Rhine 3–4 and Aach (Fig. 2), all located in the vicinity of Lake Constance (Fig. 1), it is more likely that it reflects a legacy of exchange during glacial retreat or that the ongoing drainage capture through the Danube Sinkhole plays a significant role, because water from the Danube enters the Rhine drainage at the western end of Lake Constance via the Radolfzeller Aach. However, this water has to travel for several days and up to 19 km through a complex underground karst system [14], suggesting it is unpassable for fish. Thus, exchange via meltwater streams from the retreating Rhine glacier to the Danube at the end of the Würm glacial period seems the more likely explanation. Of course, an additional role of human-mediated transport, e.g. via the release of bait fish, cannot be excluded.

Finally, many chub from the river Neckar, which is part of the Rhine catchment, were also assigned with high probability to the genetic cluster associated with the Danube catchment. A possible explanation for this pattern is yet another drainage capture. The same observation was made in the bullhead (*Cottus gobio*), in which populations from particular tributaries of the Neckar also show a genetic affinity with Danubian bullheads rather than bullheads from the Rhine drainage system [34]. The authors of that study suggest that this may be related to the fact that the river Neckar gradually eroded backward during the Pleistocene, resulting in the capture of several tributaries that were previously part of the Danubian drainage system [35, 36]. A similar scenario for the chub could also help explain the relatively high mitochondrial haplotype diversity in the Neckar compared to other sites from the Rhine catchment (Fig. 1). However, this has to remain speculative for the moment and further work is required to establish whether that is indeed why chub from the river Neckar are genetically more similar to chub from the Danube than to chub from the nearest site in the Rhine.

## Conclusions

Four major European river catchments originate in close proximity around the Swiss Alps: The Rhone, the Rhine, the Danube and the Po catchment. A combined genetic and morphological analysis of chub from these catchments identifies the chub from the Po drainage, which are part of the Adriatic lineage *sensu* Durand et al. [7], as clearly distinct. Our analyses are thus in agreement with its treatment as a distinct species, the Italian chub *S. squalus*. While this is commonly (not universally) adopted [8, 9, 37], it would presumably require elevating other phylogenetic clades to species level as well to restore consistency, highlighting the need for a systematic revision of the *S. cephalus* complex. The other drainages contain European chub, *S. cephalus*, belonging to the Western lineage as defined by Durand et al. [7]. The higher mitochondrial diversity in the Danube catchment and a nuclear genetic structure coarsely reflecting catchment structure is generally consistent with the proposed scenario of a Pleistocene colonization of central Europe from east to west via the Danube [7, 8], followed by post-glacial recolonizations from multiple refugia such as the lower Danube, the lower Rhone and possibly also the Rhine. However, we found evidence that interesting additional processes have shaped the phylogeography of the European chub around the Swiss Alps. These include a watershed crossing between the upper parts of the Rhine catchment and the Lake Geneva area of the Rhone catchment, which has also been inferred from phylogeographic patterns observed in other fish species [32, 33], as well as between the Rhine and Danube catchments near Lake Constance, which has also been inferred previously for perch [11]. An important new finding of the present study was the discovery of the predominant European chub haplotype from the Rhone and Rhine drainages in some Italian chub from southern Switzerland. This is difficult to explain by any process other than human-mediated transport. Although we do not currently have any evidence for that in the Italian chub, there are deterrent examples of endemic fish species losing their ‘identity’ by hybridization, e.g. the Italian barbel *Barbus plebejus* suffering from introgressive hybridization with introduced *B. barbus* [38], or Adriatic trout getting dissolved in large populations of stocked Atlantic trout [39]. The desirable preservation of distinct evolutionary lineages will thus require the prevention of human translocations.

## Methods

### Fish samples

Chub were collected from a total of 23 sites, three from the Danube (sites nr. 1–3), 11 from the Rhine (sites nr. 4–14), six from the Rhone (sites nr. 15–20) and three from the Po catchment (sites nr. 21–23) (Fig. 1, Additional file 1: Figure S1). Fish were caught by electro fishing with a backpack generator (FEG 1700, EFKO comm., Leutkirch, Germany) or with rod and line. The adult fish designated for morphometric analysis were anesthetized with clove oil and killed according to the animal protection laws by gill cut before photographing and taking fin clips as tissue samples. Juvenile fish were fin clipped (approx. 1 mm^2^) for genotyping and released thereafter. Fin clips were stored in 99 % ethanol until DNA extraction.

### Genotyping

#### DNA extraction

We used the salting-out DNA extraction protocol developed by Sunnucks and Hales [40], adapted to a 96 deep well plate format. Fin clips were first air dried in 8-strip microtubes. Thereafter, 300 μl of TNES buffer (50 mM Tris, pH 7.5, 400 mM NaCl, 20 nM EDTA, 0.5 % SDS) and 5 μl of 10 mg/ml proteinase K (Roche Inc., Basel, Switzerland) was added, followed by incubation at 55 °C on a shaker (Thermomixer Comfort, Eppendorf Inc., Hamburg, Germany) for 60 min at 300 rpm. Protein precipitation was performed by adding 85 μl of 5 M NaCl and shaking for 10 s. After the proteins were pelleted in a centrifuge at 4700 rpm for 10 min (Heraeus Megafuge 40R, Thermo Fisher Scientific inc, Waltham, MA, USA), the clear supernatants were transferred into a 96 deep well block. The DNA was precipitated by adding 400 μl of ice cold 100 % ethanol and pelleted by centrifugation for 10 min at 4700 rpm. The DNA pellet was washed with 700 ml of 70 % ethanol and air dried. For storage at −20 °C the DNA was resuspended in 100 μl of 1× TE buffer (100 mM Tris–HCl, 10 mM EDTA).

#### Microsatellite genotyping

Two multiplex PCR reactions were used to genotype the individuals at ten microsatellite loci: LceA149, LceC1, LceCb [41], N7G5, N7K4 [42] and LC128, LC27, LC290, LC32, LC93 [43]. Amplifications were performed in a total reaction volume of 10 μl, with primer concentrations and cycling conditions as described in [44].

#### Mitochondrial DNA

The phylogenetic relationships among different chub populations were investigated by sequencing parts of two mitochondrial genes, the cytochrome oxidase subunit I gene (COI) and cytochrome b (Cyt b). To amplify COI we used primer pair FishF1 and FishR1 [45] and for Cyt b we used primer pair Glu and Thr [46]. PCR conditions were as previously described [9] and for PCR cleanup, the NucleoSpin® Gel and PCR Clean-up kit (Macherey-Nagel, Düringen, Germany) was used according to the manufacturer’s instructions. PCR products were sequenced in both directions using the BigDye Terminator V3.1 Cycle Sequencing kit (Applied Biosystems, Foster City, CA, USA) and an ABI 3130 capillary sequencer (Applied Biosystems, Foster City, CA, USA). Sequences were edited with Geneious 6.0 (Biomatters, Auckland, New Zealand).

### Genetic analysis

#### Genetic diversity indices

Observed heterozygosity (*H*_*O*_), expected heterozygosity (*H*_*E*_) and standardized allelic richness (*AR*) at microsatellite loci as well as pairwise *F*_*ST*_ among catchments was calculated with FSTAT 2.9.4 [47]. Differentiation among catchments was also estimated with Jost’s [16] differentiation estimator (*D*_*est*_), using the R package DEMEtics [48]. The software MicroChecker [49] was used to test for the presence of null alleles at the microsatellite loci. Missing data were negligible (four missing entries in the 167 individuals × 8 loci data matrix). By pooling all sites within a catchment we achieved sufficient sample sizes but thereby ignored potential substructure within these catchments, possibly violating assumptions of Hardy-Weinberg and linkage equilibrium. We used the tests implemented in FSTAT to assess if and to what extent these assumptions were indeed violated.

#### Bayesian clustering

Microsatellite genotypes were subjected to a Bayesian Clustering analysis using the Markov chain Monte Carlo (MCMC) approach developed by Pritchard et al. [17] and implemented in Structure 2.3.4 [17]. We used the admixture model with uninformative priors. Forty simulations for each number of genetic clusters (*K*) from *K* =1 to *K* = 10 were run with a burn-in of 50’000 iterations followed by 300’000 iterations. The most likely number of genetic clusters was inferred according to the method of Evanno et al. [18], using Structure Harvester [50]. The 40 runs for the best-supported values of *K* were averaged using CLUMPP 1.1.2 [51] for visualization with the software DiSTRUCT [52].

#### Hybrid detection

Microsatellite genotypes were also used for detection of potential hybrids among chub from the Po catchment. The algorithm for this MCMC approach was developed by Anderson and Thompson [19] and implemented in the program NewHybrids 1.1 Beta3 (available from http://ib.berkeley.edu/labs/slatkin/eriq/software/software.htm). The analysis was run without prior information as well as with priors, for the latter we assigned all individuals from Rhine, Rhone and Danube as pure parental species, because we had no evidence for introgression of Italian chub mitochondrial haplotypes in the Rhine, Rhone and Danube drainages. The simulation was run with a burn in of 50’000 and followed by 300’000 iterations.

#### Phylogenetic analysis

Sequences of COI (618 bp) and Cyt b (1108 bp) were aligned using Clustal W in MEGA 6 [53]. All sequences were indel-free and have been deposited in GenBank (accession nrs KU302616 - KU302623, KU302625 - KU302642). Because preliminary tree reconstruction from COI and Cyt b produced very similar results, the sequences of both genes were concatenated. Their evolutionary relationships were inferred by constructing a Maximum Likelihood tree based on the Tamura-Nei model [54] with 1000 bootstraps in MEGA 6 [53]. The tree was rooted by including sequences from *Petroleuciscus borysthenicus* downloaded from Genbank (COI HM560281.1 and Cyt b HM560111.1 from the same voucher specimen and from *Rutilus rutilus* (accession nrs. KU302624, KU302643).

For comparability with earlier work inferring species status of fishes based on the DNA barcoding gene COI [45, 55], we also calculated pairwise haplotype differences just for COI using the Kimura 2-parameter model [15] which is the best model for sequences containing transitional an transversional substitutions.

### Morphometrics

#### Procrustes-based geometric morphometrics

Photographs of fish used in morphometric analyses were taken at a fixed distance and identical settings with a Nikon D5000 camera mounted on a tripod. Fish were placed on a white background with a fixed scale. Each fish was photographed twice but in the end one photograph was chosen at random because all were of sufficient quality. On these images we digitized 11 landmarks: tip of snout, posterior end of maxillary, posterior end of gills, posterior end of head, anterior insertion of fins (pectoral, pelvic, anal and dorsal) and superior and inferior insertion of caudal fin. Photographs were processed in a random order using TPSutil [56] for creating the input file to the landmark editor program TPS2 [57]. Shape variation was analyzed in the software MorphoJ 2.03b [53] using a full Procrustes fit to remove variation in scale, position and orientation. To correct for allometric differences, size correction was done by using the residuals of a regression of the Procrustes coordinates on log-transformed centroid size. Size-corrected shape differences among chub from the different drainages were analyzed using a canonical variate analysis (CVA), expressed as Procrustes distances and compared with permutation tests as implemented in MorphoJ.

#### Traditional morphometrics

The measurements taken from each fish were a selection of morphological features previously presented [10]. From the left side, the following measurements were taken: standard length, total length, predorsal length, postdorsal length, head length, dorsal head length, prepelvic length, preanal length, length of the fins (dorsal, pectoral, pelvic, anal), length of base of anal and dorsal fin, length of caudal peduncle, depth of caudal peduncle, snout length, eye diameter, postorbital length and interorbital width. All measurements were standardized as proportions of the standard length. Allometric changes were again corrected for by obtaining the residuals from a regression of the standardized measurements on size following the recommendations of Reist [58]. We also counted the total number of rays of all fins, not discriminating between soft rays, branched rays and spiny rays. The last double ray of the dorsal and anal fin was counted as 1.5. The scales along the lateral line were also counted. Fin color was recorded as red (=0), black (=1) or mixed (=0.5). A Principal Components Analysis (PCA) executed in R [59] was used to reduce the dimensionality of the morphometric variables before comparisons. The PCs cumulatively explaining 80 % of the variance were retained for between-catchment comparisons of fish by MANOVAs on the scores of these PCs, carried out with the statistical software R [59].

## Additional files

Additional file 1: Figure S1. Separate phylogenetic trees for each of the two mitochondrial gene sequences used (Cyt b and COI). (PDF 133 kb) Additional file 2: Table S1. Overview of sampling sites with coordinates and sample sizes used for nuclear genetic, mitochondrial and morphological analyses. (PDF 99 kb) Additional file 3: Figure S2. Excerpts of the output from STRUCTURE HARVESTER (Earl and Vonholdt 2012) employing the method of Evanno et al. (2005) to infer the most probable number of genetic clusters in the microsatellite genotypes of chub analyzed in this study. (PDF 216 kb) Additional file 4: Table S2. Catchment means (± SE) of metric, meristic and qualitative morphological traits used for traditional morphometric analysis. (PDF 81 kb)

## References

[1] Hofreiter M, Stewart J (2009). Ecological Change, Range Fluctuations and Population Dynamics during the Pleistocene. Curr Biol.

[2] Hewitt GM (1999). Post-glacial re-colonization of European biota. Biol J Linnean Soc.

[3] Reyjol Y, Hugueny B, Pont D, Bianco PG, Beier U, Caiola N, Casals F, Cowx I, Economou A, Ferreira T (2007). Patterns in species richness and endemism of European freshwater fish. Glob Ecol Biogeogr.

[4] Ribeiro F, Leunda PM (2012). Non-native fish impacts on Mediterranean freshwater ecosystems: current knowledge and research needs. Fisheries Manag Ecol.

[5] Durand JD, Unlu E, Doadrio I, Pipoyan S, Templeton AR (2000). Origin, radiation, dispersion and allopatric hybridization in the chub *Leuciscus cephalus*. Proc R Soc B Biol Sci.

[6] Banarescu P (1992). Zoogeography of Fresh Waters: Distribution and Dispersal of Freshwater Animals in North America and Eurasia.

[7] Durand JD, Persat H, Bouvet Y (1999). Phylogeography and postglacial dispersion of the chub (*Leuciscus cephalus*) in Europe. Mol Ecol.

[8] Seifertova M, Bryja J, Vyskocilova M, Martinkova N, Simkova A (2012). Multiple Pleistocene refugia and post-glacial colonization in the European chub (*Squalius cephalus*) revealed by combined use of nuclear and mitochondrial markers. J Biogeogr.

[9] Perea S, Boehme M, Zupancic P, Freyhof J, Sanda R, Ozulug M, Abdoli A, Doadrio I (2010). Phylogenetic relationships and biogeographical patterns in Circum-Mediterranean subfamily Leuciscinae (Teleostei, Cyprinidae) inferred from both mitochondrial and nuclear data. BMC Evol Biol.

[10] Kottelat M, Freyhof J. Handbook of European freshwater fishes. Cornol: Publications Kottelat; 2007: 646.

[11] Behrmann-Godel J, Gerlach G, Eckmann R (2004). Postglacial colonization shows evidence for sympatric population splitting of Eurasian perch (*Perca fluviatilis* L.) in Lake Constance. Mol Ecol.

[12] Keller O, Krayss E (2000). Die Hydrographie des Bodenseeraums in Vergangenheit und Gegenwart. Berichte der St Gallischen Naturwissenschaftlichen Gesellschaft.

[13] Rutte E. Rhein Main Donau: Wie - wann - warum sie wurden. Eine geologische Geschichte: Sigmaringen: Jan Thorbecke Verlag Sigmaringen; 1987.

[14] Hötzl H (1996). Origin of the Danube-Aach system. Environ Geol.

[15] Kimura M (1980). A simple method of estimating evolutionary rate of base substitution through comparative studies of nucleotide sequences. J Mol Evol.

[16] Jost L (2008). *G*_*ST*_ and its relatives do not measure differentiation. Mol Ecol.

[17] Pritchard JK, Stephens M, Donnelly P (2000). Inference of population structure using multilocus genotype data. Genetics.

[18] Evanno G, Regnaut S, Goudet J (2005). Detecting the number of clusters of individuals using the software STRUCTURE: a simulation study. Mol Ecol.

[19] Anderson EC, Thompson EA (2002). A model-based method for identifying species hybrids using multilocus genetic data. Genetics.

[20] Hebert PDN, Cywinska A, Ball SL, DeWaard JR (2003). Biological identifications through DNA barcodes. Proc R Soc B Biol Sci.

[21] Nichols R (2001). Gene trees and species trees are not the same. Trends Ecol Evol.

[22] Balloux F (2010). The worm in the fruit of the mitochondrial DNA tree. Heredity.

[23] Doadrio I, Kottelat M, de Sostoa A (2007). *Squalius laietanus*, a new species of cyprinid fish from north-eastern Spain and southern France (Teleostei : Cyprinidae). Ichthyol Explor Freshwat.

[24] Kottelat M, Economidis PS (2006). *Squalius orpheus*, a new species of cyprinid fish from Evros drainage, Greece (Teleostei : Cyprinidae). Ichthyol Explor Freshwat.

[25] Kottelat M, Freyhof J (2007). Handbook of European Freshwater Fishes.

[26] Pamilo P, Nei M (1988). Relationships between gene trees and species trees. Mol Biol Evol.

[27] Gherardi F, Bertolino S, Bodon M, Casellato S, Cianfanelli S, Ferraguti M, Lori E, Mura G, Nocita A, Riccardi N (2008). Animal xenodiversity in Italian inland waters: distribution, modes of arrival, and pathways. Biol Invasions.

[28] Vähä JP, Primmer CR (2006). Efficiency of model-based Bayesian methods for detecting hybrid individuals under different hybridization scenarios and with different numbers of loci. Mol Ecol.

[29] Denys GPJ, Dettai A, Persat H, Doadrio I, Cruaud C, Keith P. Status of the Catalan chub *Squalius laietanus* (Actinopterygii, Cyprinidae*)* in France: input from morphological and molecular data. Knowl Manage Aquat Ecosystems 2013(408)

[30] Freyhof, J. 2011. Squalius squalus. The IUCN Red List of Threatened Species 2011: e.T135619A4163501. http://dx.doi.org/10.2305/IUCN.UK.2008.RLTS.T135619A4163501.en.

[31] Allendorf FW (1986). Genetic drift and the loss of alleles versus heterozygosity. Zoo Biol.

[32] Largiadèr CR, Hefti D, BAFU, Mitteilung zur Fischerei (2002). Genetische Aspekte des Schutzes und der nachhaltigen Bewirtschaftung von Fischarten. Vollzug Umwelt.

[33] Vonlanthen P, Excoffier L, Bittner D, Persat H, Neuenschwander S, Largiader CR (2007). Genetic analysis of potential postglacial watershed crossings in Central Europe by the bullhead (*Cottus gobio* L.). Mol Ecol.

[34] Riffel M, Schreiber A (1995). Coarse-grained population structure in Central European sculpin (*Cottus gobio* L): Secondary contact or ongoing genetic drift?. J Zool Syst Evol Res.

[35] Hantke R (1993). Flussgeschichte Mitteleuropas.

[36] Mader M (1978). Die Flussgeschiche des Neckars und das Wandern des Albtraufs. Veröffentlichungen für Naturschutz und Landschaftspflege in Baden-Württemberg.

[37] Tancioni L, Russo T, Cataudella S, Milana V, Hett AK, Corsi E, Rossi AR (2013). Testing species delimitations in four Italian sympatric Leuciscine fishes in the Tiber River: A combined morphological and molecular approach. Plos One.

[38] Meraner A, Venturi A, Ficetola GF, Rossi S, Candiotto A, Gandolfi A (2013). Massive invasion of exotic *Barbus barbus* and introgressive hybridization with endemic *Barbus plebejus* in Northern Italy: where, how and why?. Mol Ecol.

[39] Meraner A, Gratton P, Baraldi F, Gandolfi A (2013). Nothing but a trace left? Autochthony and conservation status of Northern Adriatic *Salmo trutta* inferred from PCR multiplexing, mtDNA control region sequencing and microsatellite analysis. Hydrobiologia.

[40] Sunnucks P, Hales DF (1996). Numerous transposed sequences of mitochondrial cytochrome oxidase I-II in aphids of the genus *Sitobion* (Hemiptera: Aphididae). Mol Biol Evol.

[41] Larno V, Launey S, Devaux A, Laroche J (2005). Isolation and characterization of microsatellite loci from chub *Leuciscus cephalus* (Pisces : Cyprinidae). Mol Ecol Notes.

[42] Mesquita N, Cunha C, Hanfling B, Carvalho GR, Ze-Ze L, Tenreiro R, Coelho MM (2003). Isolation and characterization of polymorphic microsatellite loci in the endangered Portuguese freshwater fish *Squalius aradensis* (Cyprinidae). Mol Ecol Notes.

[43] Vyskocilova M, Simkova A, Martin JF (2007). Isolation and characterization of microsatellites in *Leuciscus cephalus* (Cypriniformes, Cyprinidae) and cross-species amplification within the family Cyprinidae. Mol Ecol Notes.

[44] Gouskov A, Bitterlin L, Reyes M, Vorburger C (2016). Fish Population genetic structure shaped by hydroelectric power plants in the upper Rhine catchment. Evol Appl.

[45] Ward RD, Zemlak TS, Innes BH, Last PR, Hebert PDN (2005). DNA barcoding Australia's fish species. Philos Trans R Soc B Biol Sci.

[46] Machordom A, Doadrio I (2001). Evidence of a cenozoic Betic-Kabilian connection based on freshwater fish phylogeography (*Luciobarbus*, Cyprinidae). Mol Phylogenet Evol.

[47] Goudet J: FSTAT 2.9.3.2, a program to estimate and test gene diversities and fixation indices. Available at: http://www2.unil.ch/popgen/softwares/fstat.htm (update from Goudet 1995). 2002.

[48] Gerlach G, Jueterbock A, Kraemer P, Deppermann J, Harmand P (2010). Calculations of population differentiation based on G(ST) and D: forget G(ST) but not all of statistics!. Mol Ecol.

[49] Van Oosterhout C, Hutchinson WF, Wills DPM, Shipley P (2004). MICRO-CHECKER: software for identifying and correcting genotyping errors in microsatellite data. Mol Ecol Notes.

[50] Earl DA, Vonholdt BM (2012). STRUCTURE HARVESTER: a website and program for visualizing STRUCTURE output and implementing the Evanno method. Conserv Genet Resour.

[51] Jakobsson M, Rosenberg NA (2007). CLUMPP: a cluster matching and permutation program for dealing with label switching and multimodality in analysis of population structure. Bioinformatics.

[52] Rosenberg NA (2004). DISTRUCT: a program for the graphical display of population structure. Mol Ecol Notes.

[53] Klingenberg CP (2011). MorphoJ: an integrated software package for geometric morphometrics. Mol Ecol Resour.

[54] Tamura K, Nei M (1993). Estimation of the number of nucleotide substitutions in the control region of mitochondrial-DNA in humans and chimpanzees. Mol Biol Evol.

[55] Ward RD, Hanner R, Hebert PDN (2009). The campaign to DNA barcode all fishes, FISH-BOL. J Fish Biol.

[56] Rohlf FJ. tpsUtil, file utility program. version 1.40. Departement of Ecology and Evolution, State University of New York at Stony Brook, Stony Brook NY, USA; 2008. Available at: http://life.bio.sunysb.edu/morph.

[57] Rohlf FJ. tpsDig program, version 2.05. Departement of Ecology and Evolution, State University of New York at Stony Brook, Stony Brook NY, USA; 2005. Available at: http://life.bio.sunysb.edu/morph.

[58] Reist JD (1985). An empirical-evaluation of several univariate methods that adjust for size variation in morphometric data. Can J Zool.

[59] R-Development-Core-Team (2007). R: A language and environment for statistical computing.

