# Supplementary material for: Postglacial recolonizations, watershed crossings and human translocations shape the distribution of chub lineages around the Swiss Alps
Source: BMC Evol Biol. 2016 Sep 9;16(1):185. doi: 10.1186/s12862-016-0750-9 (PMC5017123; doi:10.1186/s12862-016-0750-9)
Supplement: Additional file 1: Figure S1. — Separate phylogenetic trees for each of the two mitochondrial gene sequences used (Cyt b and COI). (PDF 133 kb) [file 12862_2016_750_MOESM1_ESM.pdf]

Additional File 1

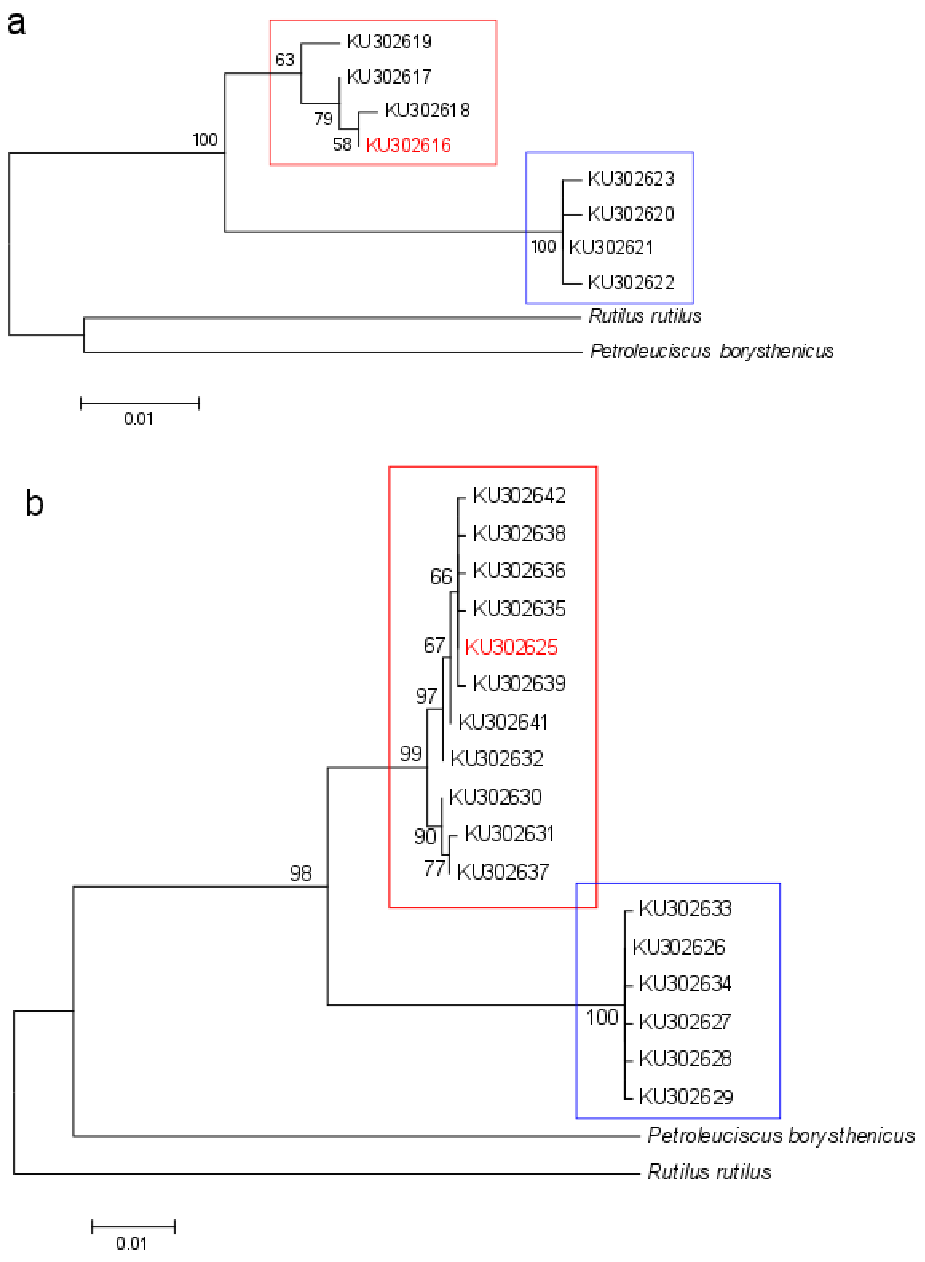

Figure S1. Separate phylogenetic trees for each of the mitochondrial gene sequences used. (a) Maximum likelihood phylogenetic tree of CO1 chub haplotypes, based on the Kimura 2-parameter model and **(b)** Cyt b chub haplotypes, based on the Tamura-Nei model. *Petroleuciscus borysthenicus* and *Rutilus rutilus* are used as outgroups. Sequences are labelled with their GenBank accession numbers. Sequences within the red rectangles belong to European chub (*Squalius cephalus*) haplotypes and those within blue rectangles to Italian chub haplotypes (*S. squalus*). Sequences belonging to concatenated Haplotype E (see Figs. 1 & 2 in main manuscript) are highlighted in red bold letters. The trees are scaled with branch lengths indicating amino acid substitutions per site (see scale bar). Numbers at nodes represent bootstrap support from 1000 replications. Lower than 50 percent support values are not shown.
