# Supplementary material for: Postglacial recolonizations, watershed crossings and human translocations shape the distribution of chub lineages around the Swiss Alps
Source: BMC Evol Biol. 2016 Sep 9;16(1):185. doi: 10.1186/s12862-016-0750-9 (PMC5017123; doi:10.1186/s12862-016-0750-9)
Supplement: Additional file 2: Table S1. — Overview of sampling sites with coordinates and sample sizes used for nuclear genetic, mitochondrial and morphological analyses. (PDF 99 kb) [file 12862_2016_750_MOESM2_ESM.pdf]

Additional file 2

Table S1. Overview of sampling sites with coordinates and and sample sizes used for nuclear genetic, mitochondrial and morphological analyses.

Nr.	Site	Coordinates WGS84	Total *N*	*N* microsatellites	N CO1 & Cyt b	N morphometrics
1	Würm	N 48° 04' 45.98" E 11° 23' 46.14"	4	4	4/4	4
2	Danube	N 48° 04' 10.35" E 09° 00' 27.61"	7	7	7/7	7
3	Günz	N 48° 15' 24.40" E 10° 19' 17.13"	5	5	5/5	5
4	Neckar	N 48° 30' 19.16" E 09° 01' 51.57"	10	10	10/10	10
5	Rhine 1	N 49° 23' 47.32" E 08° 29' 58.94"	10	10	10/10	0
6	Rhine 2	N 47° 36' 23.24" E 08° 13' 21.66"	6	6	6/6	6
7	Rhine 3	N 47° 35' 49.36" E 08° 35' 44.49"	6	5	6/6	5
8	Rhine 4	N 47° 39' 09.40" E 08° 37' 46.12"	6	6	6/6	6
9	Aach	N 47° 33' 22.20" E 09° 21' 58.62"	5	5	5/5	5
10	Lake Zurich	N 47° 12' 26.98" E 08° 46' 35.03"	5	5	5/5	5
11	Aar 1	N 47° 07' 20.34" E 07° 14' 13.74"	5	5	5/5	5
12	Aar 2	N 47° 14' 07.67" E 07° 40' 37.44"	5	5	5/5	5
13	Mentue	N 46° 47' 35.08" E 06° 44' 17.48"	7	7	7/7	7
14	Broye	N 46° 50' 02.49" E 06° 56' 05.63"	10	10	10/10	10
15	Allaine	N 47° 27' 43.15" E 07° 02' 53.35"	10	10	10/10	10
16	Doubs	N 47° 21' 36.09" E 07° 07' 22.02"	10	10	10/10	9
17	Doubs / Sâone	N 46° 54' 05.55" E 05° 04' 57.27"	10	10	10/9	0
18	Gardon	N 43° 51' 10.39" E 04° 36' 09.35"	8	7	7/7	8
19	Laire	N 46° 08' 48.44" E 05° 58' 02.95"	10	10	10/10	10
20	Venoge	N 46° 31' 04.47" E 06° 32' 39.08"	10	10	10/10	10
21	Faloppia	N 45° 49' 53.84" E 09° 00' 42.76"	2	2	2/2	2
22	Canale p.s. sinistra	N 46° 09' 08.92" E 08° 52' 47.76"	6	5	6/6	6
23	Scairolo	N 45° 56' 59.70" E 08° 54' 20.75"	13	13	13/13	10
	Total		170	167	169/168	145
