# Supplementary material for: Postglacial recolonizations, watershed crossings and human translocations shape the distribution of chub lineages around the Swiss Alps
Source: BMC Evol Biol. 2016 Sep 9;16(1):185. doi: 10.1186/s12862-016-0750-9 (PMC5017123; doi:10.1186/s12862-016-0750-9)
Supplement: Additional file 3: Figure S2. — Excerpts of the output from STRUCTURE HARVESTER (Earl and Vonholdt 2012) employing the method of Evanno et al. (2005) to infer the most probable number of genetic clusters in the microsatellite genotypes of chub analyzed in this study. (PDF 216 kb) [file 12862_2016_750_MOESM3_ESM.pdf]

Additional file 3

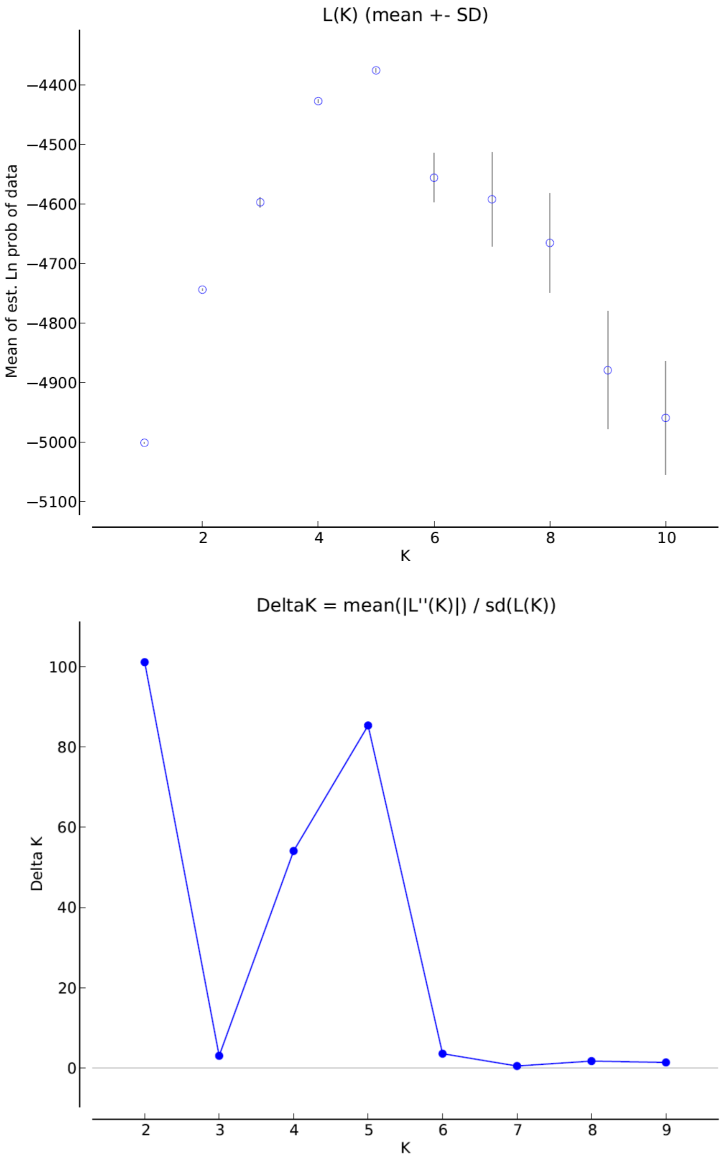

K	Reps	Mean LnP(K)	Stdev LnP(K)	Ln'(K)	|Ln''(K)|	Delta K
1	40	-5001.015000	0.495906	—	—	—
2	40	-4743.600000	1.093336	257.415000	110.600000	101.158247
3	40	-4596.785000	7.675287	146.815000	23.137500	3.014545
4	40	-4426.832500	2.185311	169.952500	118.200000	54.088416
5	40	-4375.080000	2.719559	51.752500	232.157500	85.365869
6	40	-4555.485000	40.693120	-180.405000	144.115000	3.541508
7	40	-4591.775000	78.114314	-36.290000	37.005000	0.473729
8	40	-4665.070000	82.732387	-73.295000	140.702500	1.700694
9	40	-4879.067500	98.466327	-213.997500	133.880000	1.359653
10	40	-4959.185000	94.111095	-80.117500	—	—

Figure S2. Excerpts of the output from STRUCTURE HARVESTER (Earl and Vonholdt 2012) employing the method of Evanno et al. (2005) to infer the most probable number of genetic clusters in the microsatellite genotypes of chub analyzed in this study. The mean likelihoods (from 40 replicate runs) show a unimodal distribution with a maximum at K = 5 (top panel), suggesting five as the most likely number of genetic clusters, which is also supported by a high ΔK value (bottom panel), although ΔK is even higher for K = 2, despite this number having the lowest likelihood of all values tested.
