# Supplementary material for: Postglacial recolonizations, watershed crossings and human translocations shape the distribution of chub lineages around the Swiss Alps
Source: BMC Evol Biol. 2016 Sep 9;16(1):185. doi: 10.1186/s12862-016-0750-9 (PMC5017123; doi:10.1186/s12862-016-0750-9)
Supplement: Additional file 4: Table S2. — Catchment means (± SE) of metric, meristic and qualitative morphological traits used for traditional morphometric analysis. (PDF 81 kb) [file 12862_2016_750_MOESM4_ESM.pdf]

Additional file 4

Table S2. Catchment means (± SE) of metric, meristic and qualitative morphological traits used for traditional morphometric analysis. Metric traits are presented as proportions of the standard length. Fin color was recorded as red (= 0), black (= 1) or mixed (= 0.5).

Trait	Rhine	Rhone	Danube	Po
predorsal length	0.53 ± 0.002	0.54 ± 0.002	0.54 ± 0.003	0.53 ± 0.005
postdorsal length	0.37 ± 0.002	0.37 ± 0.002	0.36 ± 0.003	0.38 ± 0.002
head length	0.25 ± 0.001	0.25 ± 0.001	0.26 ± 0.002	0.26 ± 0.003
dorsal head length	0.17 ± 0.001	0.17 ± 0.001	0.16 ± 0.001	0.16 ± 0.002
prepelvic length	0.48 ± 0.002	0.49 ± 0.002	0.49 ± 0.004	0.48 ± 0.004
preanal length	0.70 ± 0.002	0.70 ± 0.003	0.71 ± 0.003	0.69 ± 0.004
lenght of dorsal fin	0.17 ± 0.001	0.17 ± 0.002	0.18 ± 0.002	0.17 ± 0.002
length of pectoral fin	0.17 ± 0.001	0.17 ± 0.002	0.17 ± 0.003	0.17 ± 0.002
length of pelvic fin	0.15 ± 0.001	0.14 ± 0.001	0.15 ± 0.002	0.14 ± 0.002
length of anal fin	0.14 ± 0.001	0.14 ± 0.002	0.14 ± 0.003	0.13 ± 0.003
length of base of anal fin	0.10 ± 0.001	0.09 ± 0.001	0.10 ± 0.001	0.11 ± 0.002
length of base of dorsal fin	0.10 ± 0.001	0.10 ± 0.001	0.10 ± 0.002	0.10 ± 0.002
lenght of caudal peduncle	0.22 ± 0.001	0.22 ± 0.002	0.21 ± 0.003	0.22 ± 0.002
depth of caudal peduncle	0.10 ± 0.001	0.10 ± 0.001	0.10 ± 0.002	0.09 ± 0.002
snout length	0.06 ± 0.001	0.06 ± 0.001	0.06 ± 0.002	0.07 ± 0.001
eye diameter	0.05 ± 0.001	0.05 ± 0.001	0.04 ± 0.001	0.04 ± 0.001
postorbital length	0.15 ± 0.001	0.15 ± 0.002	0.15 ± 0.002	0.15 ± 0.002
interorbital width	0.10 ± 0.001	0.10 ± 0.001	0.10 ± 0.001	0.09 ± 0.002
dorsal rays	9.53 ± 0.031	9.50 ± 0.043	9.50 ± 0.000	9.50 ± 0.000
anal rays	9.48 ± 0.027	9.48 ± 0.021	9.50 ± 0.000	10.44 ± 0.098
pectoral rays	17.49 ± 0.082	17.27 ± 0.085	17.19 ± 0.209	17.06 ± 0.104
pelvic rays	9.06 ± 0.038	9.02 ± 0.038	9.00 ± 0.000	9.06 ± 0.056
caudal rays	18.98 ± 0.027	18.98 ± 0.022	19.00 ± 0.000	19.11 ± 0.076
lateral line scales	45.54 ± 0.114	45.55 ± 0.145	45.13 ± 0.328	44.94 ± 0.243
fin color	0.00 ± 0.000	0.00 ± 0.000	0.00 ± 0.000	0.86 ± 0.068
